# Long-term Clinical Outcomes After Hospitalization for Acute Respiratory Illness Due to Respiratory Syncytial Virus

**DOI:** 10.1093/cid/ciaf710

**Published:** 2025-12-23

**Authors:** David Singer, Yan Wang, Elizabeth M La, Susan I Gerber, Aozhou Wu, Keith A Betts

**Affiliations:** GSK, Philadelphia, Pennsylvania, USA; Analysis Group, Los Angeles, California, USA; GSK, Philadelphia, Pennsylvania, USA; GSK, Philadelphia, Pennsylvania, USA; Analysis Group, Los Angeles, California, USA; Analysis Group, Los Angeles, California, USA

**Keywords:** respiratory syncytial virus, acute respiratory illness, older adults, hospitalization, clinical outcomes

## Abstract

**Background:**

Acute respiratory illnesses (ARIs) can be severe in older adults and adults with chronic conditions. We compared long-term outcomes of United States patients aged ≥50 years with ≥1 hospitalized ARI due to respiratory syncytial virus (RSV-ARI cohort) versus controls without ARI (control cohort) and patients with ≥1 hospitalized influenza-ARI (influenza-ARI cohort).

**Methods:**

This retrospective study used 1 October 2015–30 June 2023 claims data to evaluate clinical outcomes across the three cohorts. The index date was defined as the start of an ARI episode. Cumulative incidence functions assessed risks of readmission, all-cause mortality, myocardial infarction (MI), asthma and chronic obstructive pulmonary disease (COPD) exacerbation, and hospitalization due to heart failure (HHF); adjusted risks were compared using multivariable regression models.

**Results:**

A total of 14 759, 77 468, and 73 795 patients were selected into the RSV-ARI, influenza-ARI, and control cohorts, respectively. The RSV-ARI cohort had a substantially higher adjusted risk of all-cause mortality than controls, with the highest adjusted hazard ratio (95% confidence interval) 0–30 days post-index (10.772 [9.190, 12.627]). MI risk followed a pattern similar to all-cause mortality. Adjusted risks of asthma exacerbation, COPD exacerbation, and HHF were significantly higher in the RSV-ARI cohort than controls, and similar between RSV-ARI and influenza-ARI cohorts.

**Conclusions:**

RSV-ARI had considerable long-term impact on clinical outcomes, with measurable increases in outcomes associated with RSV-ARI when compared with controls, and similar outcomes compared to influenza-ARI. These findings can inform RSV prevention efforts and support future research on the long-term impact of RSV.

## Teaser

Risks of adverse clinical outcomes, controlling for baseline characteristics, among adults aged ≥50 years hospitalized with respiratory syncytial virus-acute respiratory illness (ARI) were substantially higher than in controls with no recent ARI, and similar to those hospitalized with influenza-ARI.

Respiratory syncytial virus (RSV) is a common virus that causes acute respiratory illness (ARI), often resulting in mild, cold-like symptoms in adults. However, RSV can result in severe outcomes, especially in older adults and adults with chronic cardiovascular or pulmonary conditions, weakened immune systems, and certain other medical conditions (eg, diabetes) [1, 2]. A recent modeling study estimated that without RSV vaccination, 177 822 hospitalizations and 14 249 deaths occur annually due to RSV among adults aged ≥60 years [3]. Another study reported a 5.6% in-hospital mortality rate among adults aged ≥60 years hospitalized with RSV infection [4].

Previous literature has demonstrated impacts of RSV on healthcare resource utilization (HRU). A systematic review of medically-attended RSV-associated illness among adults aged ≥65 years estimated, after adjustment for RSV under-detection due to test sensitivity, an adjusted annual incidence of 267 hospitalizations per 100 000 people [5]. RSV infection can also exacerbate existing comorbidities and impact functional status post-acute illness, with declines in daily activities and quality of life (QoL) reported in a three-year prospective study [6], highlighting the importance of understanding longer-term impacts of RSV illness. However, current evidence on longer-term RSV-ARI outcomes among adults aged ≥50 years is limited, with insufficient research comparing the impact of RSV-ARI hospitalization to an appropriate comparator population representing the long-term health outcomes these patients would have experienced without severe RSV disease.

This study aimed to describe and compare outcomes of United States (US) patients aged ≥50 years who had RSV-ARI with ≥1 hospitalization during the ARI episode to a control group with no ARI and to patients who had influenza-ARI with ≥1 hospitalization during the ARI episode. Findings from this study can inform RSV prevention efforts and support future evaluations of the impact of RSV on health outcomes.

## METHODS

### Study Design, Population, and Data Source

This was a retrospective, observational study utilizing Optum^®^ Clinformatics^®^ Data Mart, which includes claims data from patients enrolled in commercial or Medicare Advantage insurance plans. Empirical algorithms identified patients with RSV and patients with influenza and defined the period of an ARI episode. A medically-attended ARI episode was defined as a period with ≥1 inpatient, emergency department, or outpatient encounter with ARI diagnosis (Supplementary Appendix A). Data from 1 October 2015–30 June 2023 were used; the identification period for RSV- and influenza-ARI episodes was 1 October 2016–2 June 2023.

**Figure 1.**
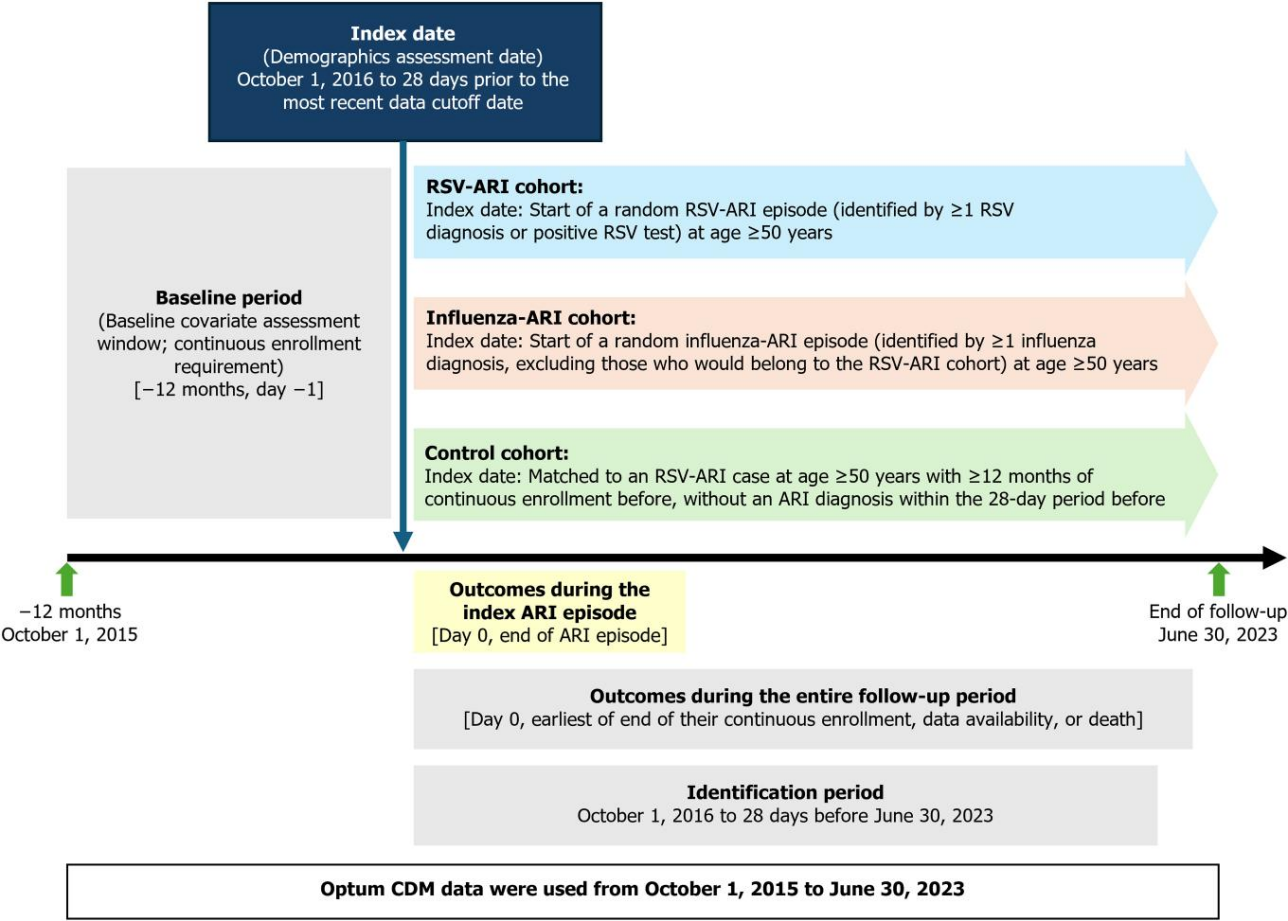
Study design schema. Abbreviations: ARI, acute respiratory illness; CDM, Clinformatics^®^ Data Mart; RSV, respiratory syncytial virus.

Three cohorts were constructed: patients aged ≥50 years who had ≥1 RSV-ARI episode involving hospitalization (RSV-ARI cohort), those who had ≥1 influenza-ARI episode involving hospitalization (influenza-ARI cohort), and those without recent ARI (control cohort). Analyses were conducted as comparisons between the RSV-ARI cohort and the control cohort, as well as between the RSV-ARI cohort and the influenza-ARI cohort. For the control cohort, five patients with no recent ARI of any cause were matched with each patient in the RSV-ARI cohort based on index date. This control comparison allowed for the evaluation of the overall disease burden caused by RSV-ARI among patients aged ≥50 years against a counterfactual representing what may be observed had RSV-ARI not occurred. Meanwhile, the influenza-ARI cohort was used as an active comparator for disease burden due to another potentially serious vaccine-preventable, viral respiratory disease (Figure 1).

#### Study Eligibility Criteria

Patients were included in the RSV-ARI or influenza-ARI cohorts if they had ≥1 RSV- or influenza-ARI episode at age ≥50 years involving ≥1 hospitalization and had continuous claims enrollment for ≥12 months before the episode's start. If an ARI episode included both RSV-ARI and influenza-ARI diagnosis codes (Supplementary Appendix B), it was classified as RSV-ARI and included in the RSV-ARI cohort only.

Patients were included in the control cohort if they had no history of an RSV- or influenza-ARI at age ≥50 years in the data cut, an eligible index date at age ≥50 years matching with a patient in the RSV-ARI cohort, ≥12 months of continuous enrollment before the index date, and no recent ARI, defined as no claims with ARI diagnosis within 28 days before the index date.

### Study Measures

This study compared (a) hospitalized RSV-ARI versus hospitalized influenza-ARI and (b) hospitalized RSV-ARI versus not having any recent ARI (controls; Supplementary Table 1). Baseline clinical and demographic characteristics were defined as those measured in the 12-month period before the index date and were assessed in each cohort.

Time-to-first-event outcomes during the follow-up period were readmission (among patients discharged alive from the first hospitalization), all-cause mortality, and myocardial infarction (MI). Risk of readmission was reported at 30 days and 3 months (91 days) following discharge from the first hospitalization of the index ARI episode; risk of all-cause mortality and MI per cohort were reported at 30 days, 3 months, 12 months, 3 years, and 5 years post-index.

Recurrent time-to-event outcomes included asthma exacerbation, chronic obstructive pulmonary disease (COPD) exacerbation, and hospitalization due to heart failure (HHF). Mean cumulative counts of these outcomes at 30 days, 3 months, 12 months, 3 years, and 5 years post-index were reported per cohort among patients with these comorbidities at baseline.

### Analytical Approach

Patient characteristics (all cohorts) and outcomes during the ARI episode (RSV- and influenza-ARI cohorts) were described using mean and standard deviation (SD) for continuous variables, and counts and proportions for categorical variables. For patient characteristics, count and proportion of missingness were summarized. Cross-cohort comparisons used Wilcoxon rank-sum tests for continuous and count variables; *χ*^2^ tests were used to compare categorical variables.

Time-to-first-event outcomes were analyzed for each cohort in the overall population and stratified by age and comorbidity subgroups defined in Supplementary Table 2. All-cause mortality was described using the Kaplan-Meier method; time to all other outcomes without death as a component was described using a cumulative incidence function with death as the competing risk. Varying-coefficient, multivariable-adjusted Cox proportional hazards models were fitted to compare each time-to-first-event outcome for RSV-ARI versus influenza-ARI cohorts and the control cohort estimating separate hazard ratios for 0–30, 31–90, 91–365, and >365-day follow-up periods, overall and by subgroup. In the presence of a competing risk event, a cause-specific hazard model was fitted.

Recurrent time-to-event outcomes per cohort and for age and comorbidity subgroups defined in Supplementary Table 3 were described using mean cumulative event functions accounting for death as a competing risk, to estimate mean cumulative counts of recurrences at 30 days, 3 months, 12 months, 3 years, and 5 years [7, 8]. Outcome risks overall and by subgroup were compared by exposure status using multivariable-adjusted, cause-specific Andersen-Gill Cox proportional hazards models to account for the recurrent nature of the outcomes by allowing individuals to contribute multiple event episodes, while accommodating variations in the at-risk periods between events.

Multivariable-adjusted regression models for both time-to-first-event and recurrent time-to-event outcomes, overall and by subgroup, controlled for demographics, temporal trends using splines, risk factors for severe RSV disease, preventive treatments, baseline HRU (Table 1), and baseline costs. These variables were adjusted for in the analyses due to their potential role as confounders, or proxies for confounders, associated with the increased risk of severe RSV disease and increased risk of the outcomes of interest; splines were utilized to account for temporal variations in these factors, due in part to the coronavirus disease 2019 (COVID-19) pandemic. For time-to-event nonmortality outcomes, death was considered a competing risk if the patient did not have an outcome of interest by imputed date of death. Otherwise, patients without the event of interest were censored by end of follow-up.

**Table 1.** **Patient Baseline Characteristics by Cohort** ^a^

	Cohort of RSV-ARI With Hospitalization N = 14 759	Cohort of Influenza-ARI With Hospitalization N = 77 468	*P* value	Control Cohort N = 73 795	*P* value
	(A)	(B)	(A) versus (B)	(C)	(A) versus (C)
**Demographics as of the index date**					
Age (y)	…	…	<.001	…	<.001
Mean ± SD	76.5 ± 9.8	75.4 ± 9.9		69.5 ± 10.4	
Age (y), n (%)	…	…	<.001	…	<.001
50–59	945 (6.40%)	6149 (7.94%)		15 372 (20.83%)	
60–64	966 (6.55%)	5703 (7.36%)		7828 (10.61%)	
65–74	3986 (27.01%)	21 955 (28.34%)		26 094 (35.36%)	
≥75	8862 (60.04%)	43 661 (56.36%)		24 501 (33.20%)	
Sex, n (%)	…	…	<.001	…	<.001
Female	8938 (60.56%)	43 296 (55.89%)		40 594 (55.01%)	
Male	5821 (39.44%)	34 163 (44.10%)		33 191 (44.98%)	
Unknown	<0.1%	<0.1%		<0.1%	
Race, n (%)	…	…	<.001	…	<.001
White	10 847 (73.49%)	54 405 (70.23%)		52 178 (70.71%)	
Black	1512 (10.24%)	9860 (12.73%)		7214 (9.78%)	
Asian	321 (2.17%)	1652 (2.13%)		2836 (3.84%)	
Hispanic	1225 (8.30%)	7261 (9.37%)		7488 (10.15%)	
Unknown	854 (5.79%)	4290 (5.54%)		4079 (5.53%)	
Geographic region of residence, n (%)	…	…	<.001	…	<.001
Northeast	2796 (18.94%)	11 367 (14.67%)		8809 (11.94%)	
Midwest	3810 (25.81%)	17 671 (22.81%)		16 260 (22.03%)	
West	3415 (23.14%)	15 027 (19.40%)		18 775 (25.44%)	
South	4729 (32.04%)	33 362 (43.07%)		29 349 (39.77%)	
Unknown	<0.1%	<0.1%		602 (0.82%)	
Duration of follow-up time (m)	…	…	<.001	…	<.001
Mean ± SD	18.8 ± 19.1	23.0 ± 21.7		26.2 ± 21.5	
Season at index, n (%)	…	…	<.001	…	1.0
Winter	8161 (55.30%)	45 691 (58.98%)		40 805 (55.30%)	
Spring	2268 (15.37%)	17 310 (22.34%)		11 340 (15.37%)	
Summer	986 (6.68%)	4051 (5.23%)		4930 (6.68%)	
Autumn	3344 (22.66%)	10 416 (13.45%)		16 720 (22.66%)	
COVID-19 period at index, n (%)	…	…	<.001	…	1.0
Before COVID-19 pandemic	8170 (55.36%)	53 797 (69.44%)		40 850 (55.36%)	
After start of the COVID-19 pandemic	6589 (44.64%)	23 671 (30.56%)		32 945 (44.64%)	
Insurance payer type at index, n (%)	…	…	<.001	…	<.001
Medicare	13 471 (91.27%)	69 450 (89.65%)		50 185 (68.01%)	
Commercial	1108 (7.51%)	7042 (9.09%)		22 386 (30.34%)	
Commercial and Medicare	167 (1.13%)	926 (1.20%)		1190 (1.61%)	
Unknown	<0.1%	<0.1%		<0.1%	
**Risk factors for serious infection during the 1-y baseline period, n (%)**					
Chronic respiratory diseases	8396 (56.89%)	37 344 (48.21%)	<.001	11 685 (15.83%)	<.001
COPD	6713 (45.48%)	29 606 (38.22%)	<.001	6949 (9.42%)	<.001
Asthma	2766 (18.74%)	11 564 (14.93%)	<.001	4328 (5.86%)	<.001
HF	5746 (38.93%)	24 067 (31.07%)	<.001	5120 (6.94%)	<.001
CAD	6000 (40.65%)	28 374 (36.63%)	<.001	10 589 (14.35%)	<.001
Cardiac arrhythmias	6306 (42.73%)	26 567 (34.29%)	<.001	9372 (12.70%)	<.001
Diabetes	6379 (43.22%)	34 253 (44.22%)	<.05	17 595 (23.84%)	<.001
Chronic kidney disease	5668 (38.40%)	26 102 (33.69%)	<.001	9705 (13.15%)	<.001
Chronic liver disease	1446 (9.80%)	6740 (8.70%)	<.001	3530 (4.78%)	<.001
Chronic cardiac diseases	9602 (65.06%)	44 218 (57.08%)	<.001	17 289 (23.43%)	<.001
Chronic cardiopulmonary diseases	12 111 (82.06%)	56 784 (73.30%)	<.001	23 794 (32.24%)	<.001
CCI^b^	…	…	<.001	…	<.001
Mean ± SD	3.3 ± 2.5	2.9 ± 2.4		1.0 ± 1.6	
Immunosuppressive conditions^c^	534 (3.62%)	1621 (2.09%)	<.001	487 (0.66%)	<.001
Prior RSV-ARI	324 (2.20%)	0	—	0	—
**Preventive measures during the 1-y baseline period, n (%)**					
Influenza vaccination	5359 (36.31%)	25 074 (32.37%)	<.001	18 787 (25.46%)	<.001
COVID-19 vaccination	542 (3.67%)	1405 (1.81%)	<.001	2358 (3.20%)	<.01
Pneumococcal vaccination	1873 (12.69%)	9357 (12.08%)	<.05	6177 (8.37%)	<.001
**HRU during the baseline period**					
Number of inpatient admissions	…	…	<.001	…	<.001
Mean ± SD	0.9 ± 1.5	0.7 ± 1.3		0.1 ± 0.5	
Number of ED visits	…	…	<.001	…	<.001
Mean ± SD	1.6 ± 2.9	1.4 ± 2.8		0.6 ± 1.6	
Number of outpatient visits	…	…	<.001	…	<.001
Mean ± SD	40.1 ± 39.3	34.3 ± 36.4		15.1 ± 20.0	
Received care at long-term care facility	1787 (12.11%)	8275 (10.68%)	<.001	1371 (1.86%)	<.001
Received care at skilled nursing facility	2652 (17.97%)	12 034 (15.53%)	<.001	2173 (2.94%)	<.001
Received home health services	7904 (53.55%)	36 397 (46.98%)	<.001	12 090 (16.38%)	<.001

Abbreviations: ARI, acute respiratory illness; CAD, coronary artery disease; CCI, Charlson Comorbidity Index; COPD, chronic obstructive pulmonary disease; COVID-19, Coronavirus Disease 2019; ED, emergency department; HF, heart failure; HIV, human immunodeficiency virus; HRU, healthcare resource utilization; m, months; RSV, respiratory syncytial virus; SD, standard deviation; y, years.

^a^Baseline characteristics were summarized for 14 759 patients in the RSV-ARI cohort and 77 468 patients in the influenza-ARI cohort with inpatient as their highest level of care in their selected ARI episode, and for 73 795 patients in the control cohort with their matched RSV-ARI patients having inpatient as the highest level of care in the selected ARI episode.

^b^The CCI was defined based on criteria by Quan (2011) [29].

^c^Immunosuppressive conditions were defined as having any of the following procedures or conditions: hematopoietic stem cell transplant, solid organ transplant, or HIV.

This study followed the Strengthening the Reporting of Observational Studies in Epidemiology (STROBE) reporting guidelines for cohort studies (Supplementary Appendix C).

## RESULTS

### Study Population and Baseline Characteristics

A total of 14 759 patients were selected into the RSV-ARI cohort, 77 468 into the influenza-ARI cohort, and 73 795 into the control cohort (Supplementary Figure 1). Baseline characteristics between the RSV-ARI and influenza-ARI cohorts were similar, with slightly more comorbidities and higher HRU in the RSV-ARI cohort. Mean (SD) ages in the RSV-ARI and influenza-ARI cohorts were higher than among controls (76.5 [9.8] and 75.4 [9.9] versus 69.5 [10.4] years, respectively). The RSV-ARI cohort had a higher mean (SD) Charlson Comorbidity Index (CCI) at 3.3 (2.5) versus 2.9 (2.4) in the influenza-ARI and 1.0 (1.6) in the control cohort (Table 1). Supplementary Table 4 summarizes outcomes occurring during ARI episodes.

### Time-to-first-event Outcomes During the Follow-up Period

#### Risk of Hospital Readmission

Hospital readmission risk was generally similar between the RSV-ARI and influenza-ARI cohorts, with a slightly higher risk (measured as event probability) among RSV-ARI patients. In the RSV-ARI cohort, hospital readmission risk (95% confidence interval [CI]) was 16.1% (15.5–16.7%) at 30 days and 26.1% (25.3–26.8%) at 3 months; in the influenza-ARI cohort, risk was 14.4% (14.1–14.6%) and 23.3% (23.0–23.6%) at 30 days and 3 months, respectively (Table 2). Readmission risk was also evaluated by age and baseline comorbidity, and was generally higher among patients with diabetes, COPD, heart failure (HF), and coronary artery disease (CAD) at baseline than in the overall sample (Supplementary Tables 5 and 6).

**Table 2.** Risk of Hospital Readmission at 30 Days and 3 Months

Cohort		Risk of Hospital Readmission at Key Time Points	
		30 Days	3 Months
RSV-ARI with hospitalization^a^ (n = 14 257)	Number at risk^b^	10 697	8694
	Cumulative number of events	2249	3603
	Event probability^c^ (95% CI)	0.161 (0.155, 0.167)	0.261 (0.253, 0.268)
Influenza-ARI with hospitalization^a^ (n = 74 930)	Number at risk^b^	57 720	48 202
	Cumulative number of events	10 581	16 961
	Event probability^c^ (95% CI)	0.144 (0.141, 0.146)	0.233 (0.230, 0.236)

Abbreviations: ARI, acute respiratory illness; CI, confidence interval; RSV, respiratory syncytial virus.

^a^Hospital readmission was evaluated among patients in the RSV-ARI and influenza-ARI cohorts who had inpatient as the highest level of care in the selected ARI episode. Five hundred and two patients in the RSV-ARI cohort and 2538 patients in the influenza-ARI cohort who died at the time of the ARI during the first hospitalization were excluded. The start date for the assessment of the risk of readmission was the discharge date of the first hospitalization during the ARI episode.

^b^Recurrent events were not considered in this analysis.

^c^The risk of hospital readmission was estimated based on the cumulative incidence function, accounting for death as a competing risk.

#### Risk of All-cause Mortality

**Figure 2.**
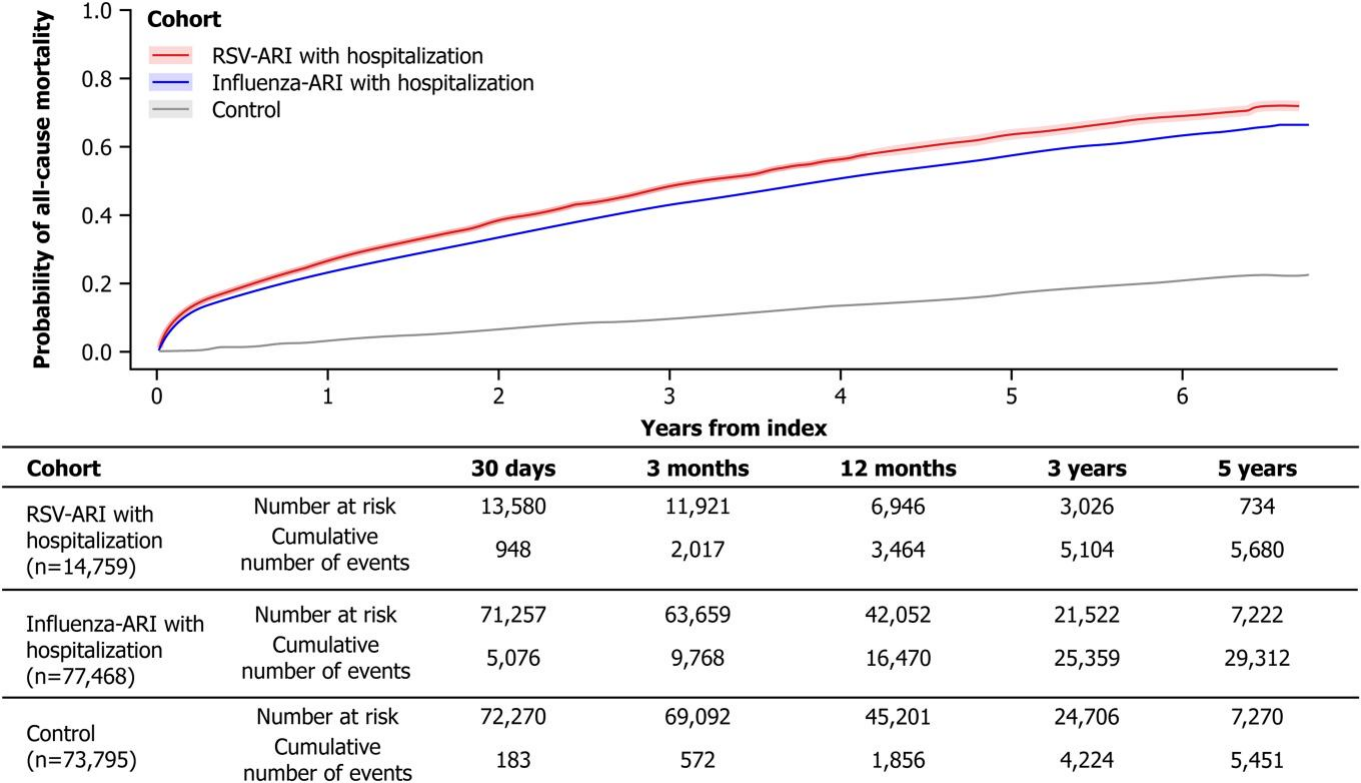
Risk of all-cause mortality over time.^a^ Abbreviations: ARI, acute respiratory illness; CI, confidence interval; RSV, respiratory syncytial virus. ^a^Curve shading indicates 95% CIs for probability.

Mortality risk was generally similar between the RSV-ARI and influenza-ARI cohorts over time, and was substantially lower among controls. At 30 days and 12 months post-index, the risk of all-cause mortality (95% CI) changed from 6.5% (6.1–6.9%) to 26.3% (25.5–27.0%) in the RSV-ARI, 6.6% (6.4–6.8%) to 23.3% (23.0–23.6%) in the influenza-ARI, and 0.3% (0.2–0.3%) to 3.1% (2.9–3.2%) in the control cohorts (Figure 2).

**Figure 3.**
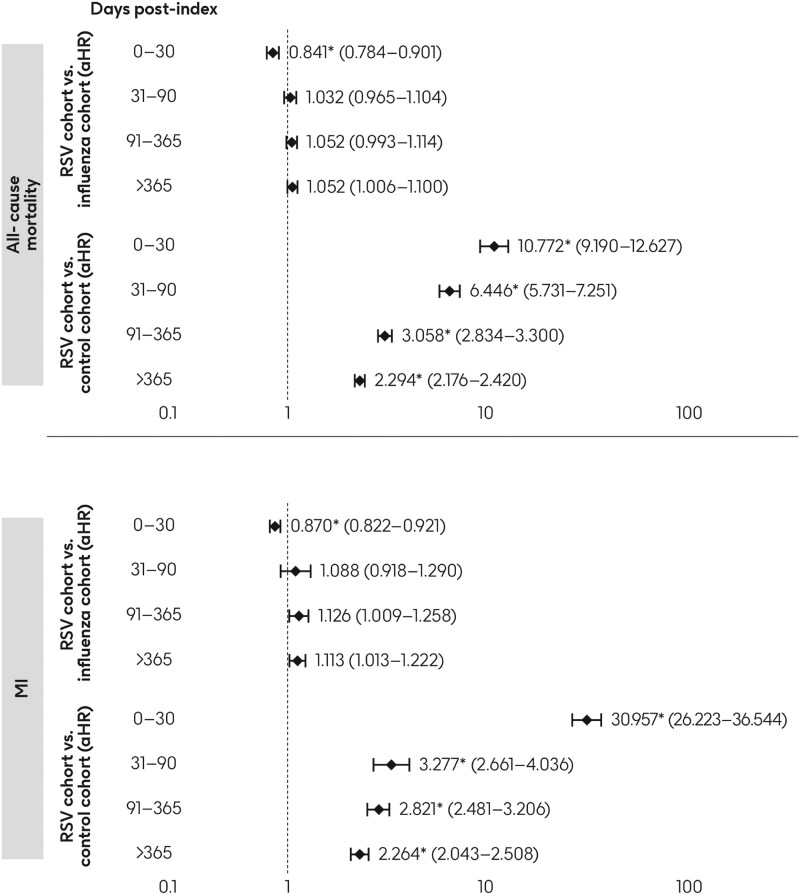
Adjusted association of all-cause mortality and MI with RSV-ARI involving hospitalization. Abbreviations: aHR, adjusted hazard ratio; ARI, acute respiratory illness; MI, myocardial infarction; RSV, respiratory syncytial virus; vs., versus. *P < .001.

In the adjusted analyses, hospitalized RSV- and influenza-ARI were associated with generally similar adjusted risks of all-cause mortality over time (adjusted hazard ratio [aHR] 0.841 [0.784–0.901] 0–30 days postindex and 1.052 [1.006–1.100] > 365 days post-index). Compared with controls, the RSV-ARI cohort had a substantially increased risk of all-cause mortality, with the highest aHR 0–30 days (10.772 [9.190–12.627]) and a significantly increased risk remaining after 365 days (2.294 [2.176–2.420] post-index; Figure 3).

#### Risk of Myocardial Infarction

**Figure 4.**
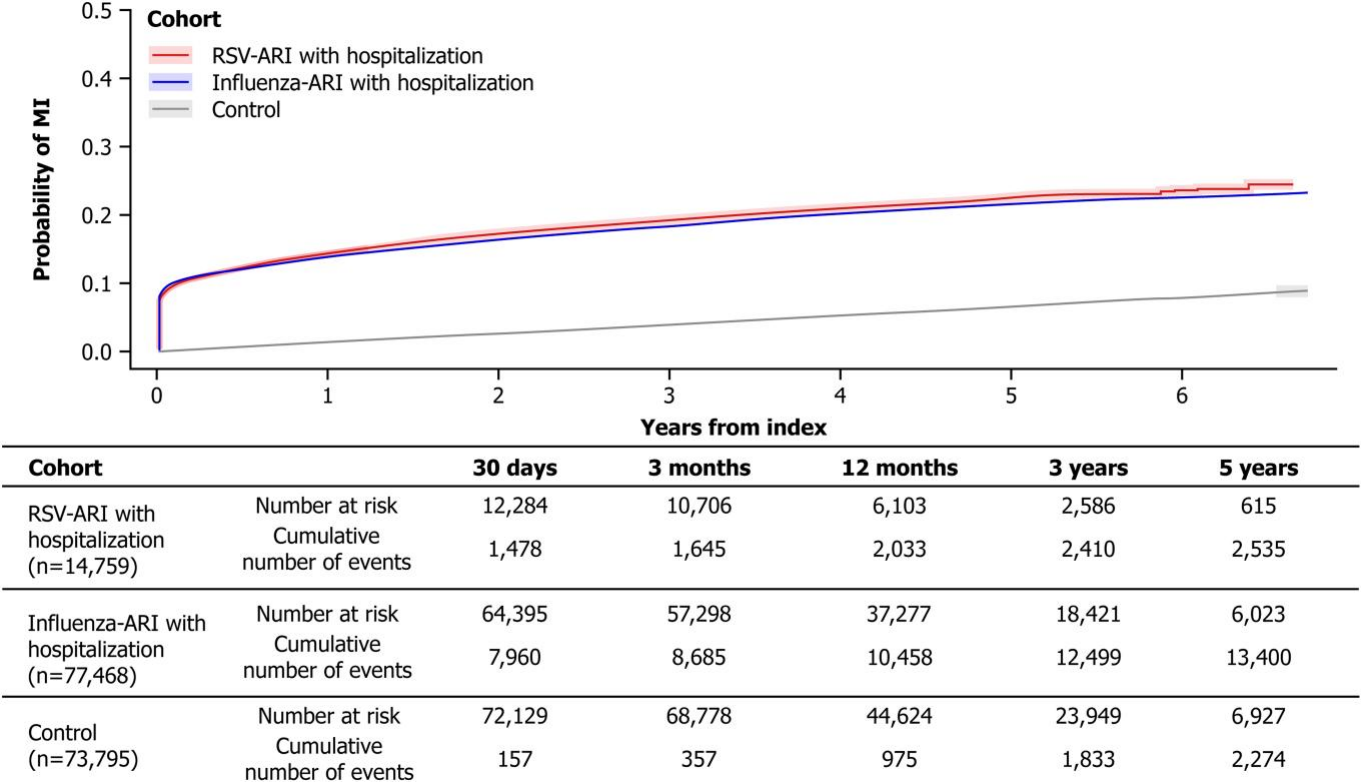
Risk of MI over time.^a^ Abbreviations: ARI, acute respiratory illness; CI, confidence interval; MI, myocardial infarction; RSV, respiratory syncytial virus. ^a^Curve shading indicates 95% CIs for probability.

MI risk over time was generally similar between RSV-ARI and influenza-ARI cohorts, compared with a substantially lower risk over time among controls. At 30 days and 12 months postindex, MI risk in the RSV-ARI, influenza-ARI, and control cohorts changed from 10.0% (9.6–10.5%) to 14.5% (13.9–15.1%), 10.3% (10.1–10.5%) to 14.0% (13.8–14.3%), and 0.2% (0.2–0.3%) to 1.6% (1.5–1.7%), respectively (Figure 4). MI risk also increased with age (Supplementary Tables 7 and 8) and was higher in patients with diabetes, COPD, HF, and CAD at baseline than in the overall sample (Supplementary Table 9).

RSV-ARI and influenza-ARI cohorts had generally similar adjusted risks of MI over time (aHR 0.870 [0.822–0.921] 0–30 days and 1.113 [1.013–1.222] > 365 days post-index). The RSV-ARI cohort had an increased adjusted risk of MI compared to controls, with the highest aHR 0–30 days (30.957 [26.223–36.544]) and a significantly increased adjusted risk even after 365 days (2.264 [2.043–2.508] post-index; Figure 3).

### Recurrent Time-to-event Outcomes During the Follow-up Period

#### Risk of Asthma Exacerbations

**Figure 5.**
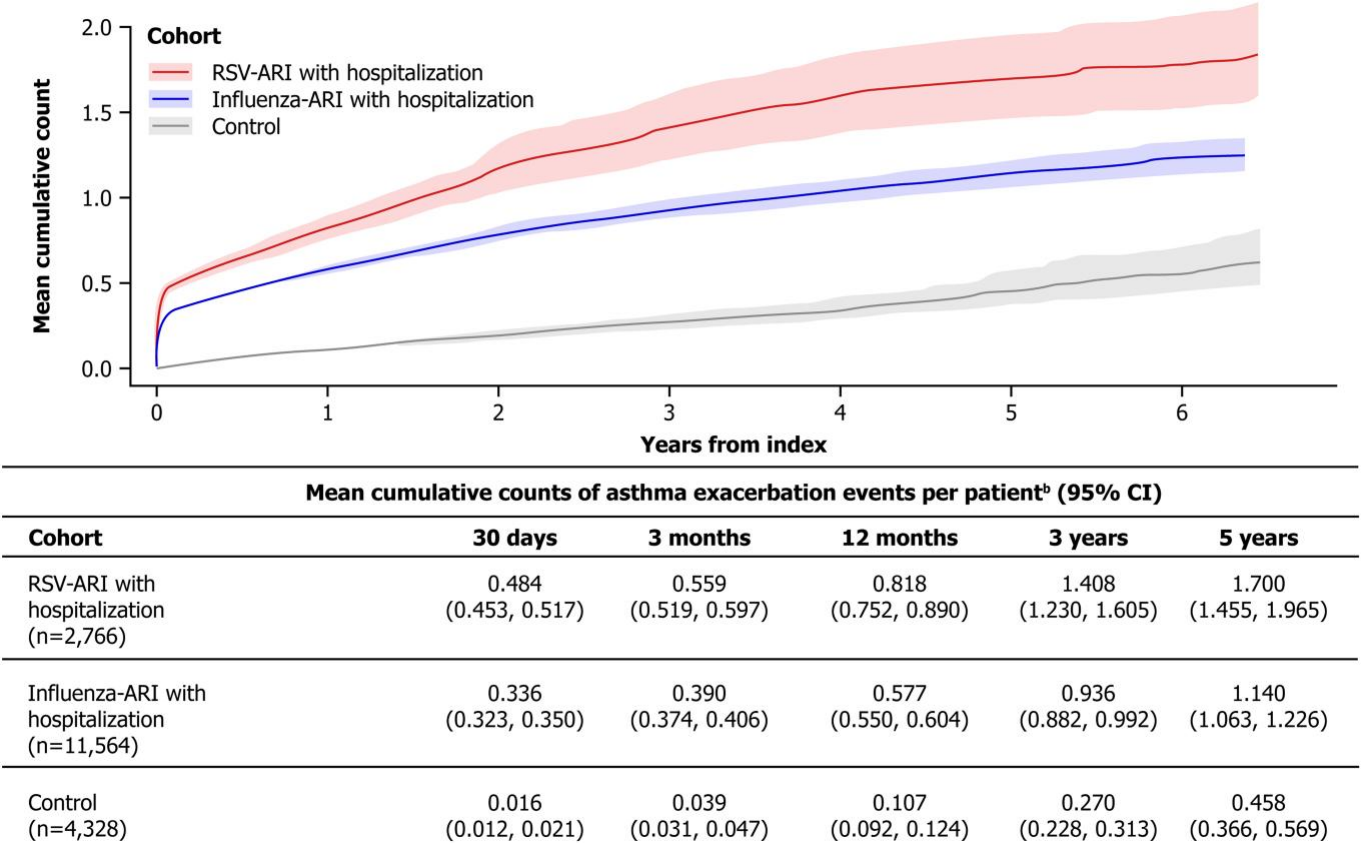
Mean cumulative counts of asthma exacerbation among people with asthma at baseline.^a^ Abbreviations: ARI, acute respiratory illness; CI, confidence interval; RSV, respiratory syncytial virus. ^a^Curve shading indicates 95% CIs for mean cumulative counts. ^b^The mean number of asthma exacerbation events per patient was estimated based on the mean cumulative count functions, accounting for death as a competing risk.

The RSV-ARI cohort had consistently higher mean cumulative counts of asthma exacerbation events than the influenza-ARI and control cohorts (Figure 5).

**Figure 6.**
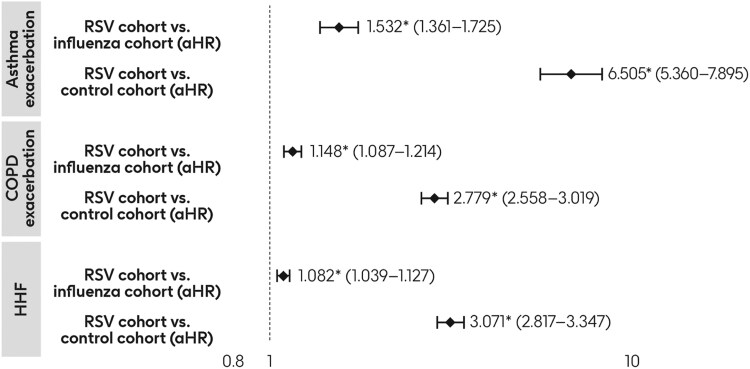
Adjusted association of recurrent time-to-event outcomes with RSV-ARI involving hospitalization among people with asthma, COPD, or HHF at baseline.^a^ Abbreviations: aHR, adjusted hazard ratio; ARI, acute respiratory illness; COPD, chronic obstructive pulmonary disease; HF, heart failure; HHF, hospitalization due to heart failure; RSV, respiratory syncytial virus; vs., versus. *P < .001. ^a^Measured among people with the respective condition at baseline.

Among people with asthma at baseline, the RSV-ARI cohort showed higher adjusted risk (aHR [95% CI]) of asthma exacerbation compared with both the influenza-ARI (1.532 [1.361–1.725]) and control cohorts (6.505 [5.360–7.895]) across ages (Figure 6).

#### Risk of Chronic Obstructive Pulmonary Disease Exacerbations

**Figure 7.**
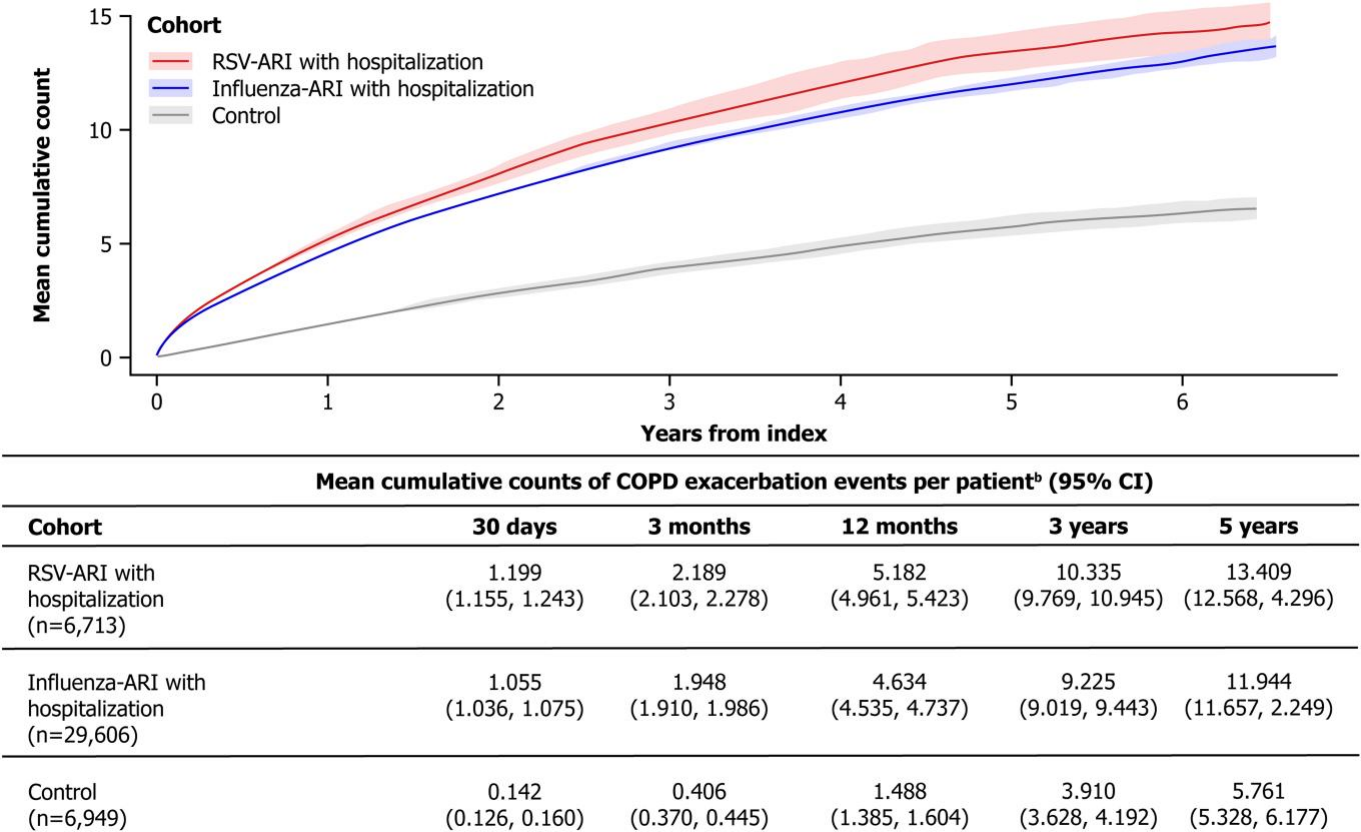
Mean cumulative counts of COPD exacerbation among people with COPD at baseline.^a^ Abbreviations: ARI, acute respiratory illness; CI, confidence interval; COPD, chronic obstructive pulmonary disease; RSV, respiratory syncytial virus. ^a^Curve shading indicates 95% CIs for mean cumulative counts. ^b^The mean number of COPD exacerbation events per patient was estimated based on the mean cumulative count functions, accounting for death as a competing risk.

Similar trends were observed for COPD exacerbation events; the RSV-ARI cohort had higher mean cumulative COPD exacerbation event counts than the influenza-ARI and control cohorts (Figure 7).

In the subgroup with COPD at baseline, the RSV-ARI cohort also consistently showed higher adjusted risk (aHR [95% CI]) of COPD exacerbation compared to influenza-ARI (1.148 [1.087–1.214]) and control (2.779 [2.558–3.019]) cohorts (Figure 6).

#### Risk of Hospitalization Due to Heart Failure

**Figure 8.**
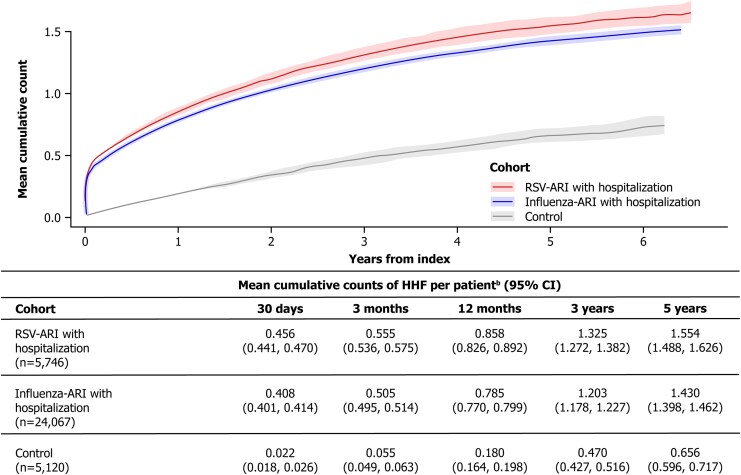
Mean cumulative counts of HHF events among people with HF at baseline.^a^ Abbreviations: ARI, acute respiratory illness; CI, confidence interval; HF, heart failure; HHF, hospitalization due to heart failure; RSV, respiratory syncytial virus. ^a^Curve shading indicates 95% CIs for mean cumulative counts. ^b^The mean number of hospitalizations due to HF per patient was estimated based on the mean cumulative count functions, accounting for death as a competing risk.

As with asthma and COPD exacerbation, RSV-ARI patients had the highest mean cumulative counts of HHF events, followed by influenza-ARI and then control cohorts (Figure 8).

RSV-ARI cohort patients with HF at baseline had consistently higher adjusted risk (aHR [95% CI]) of HHF compared to both influenza-ARI (1.082 [1.039–1.127]) and control (3.071 [2.817–3.347]) cohorts (Figure 6).

## DISCUSSION

This large, retrospective US claims database analysis provides a novel, up-to-date evaluation of the health outcomes of adults aged ≥50 years hospitalized with RSV-ARI, compared to those hospitalized with influenza-ARI or without ARI, including over longer-term follow-up.

Substantial risk of readmission was identified among adults with hospitalized RSV-ARI, and risk increased with age and presence of comorbidities, similar to RSV-associated readmission rates 1 month post-hospitalization reported previously [9, 10].

The RSV-ARI cohort also had higher all-cause mortality and MI risks than controls, with adjusted risks remaining over two-fold higher 12 months post-index in adults with hospitalized RSV-ARI episodes than those without, suggesting potential long-term impacts of RSV hospitalization beyond the substantial increased risk of these outcomes around the time of the RSV-ARI. RSV-ARI cohort mortality risk was consistent with a study among hospitalized RSV-positive adults aged ≥60 years identifying cumulative mortality rates of 8.6%, 12.3%, and 25.8% within 1, 3, and 12 months of admission, respectively [4]. Influenza-ARI cohort mortality risk at 30 days (6.6%) was also similar to previous studies [11, 12]. Another RSV study identified increasing age as an independent mortality risk factor, where older adults hospitalized for RSV had a higher risk of death within 90 days of hospitalization than patients admitted with influenza B [13]. Other studies comparing RSV- and influenza-related mortality have considered short-term outcomes and adjusted for limited numbers of covariates. One observational study reported higher risks of invasive mechanical ventilation or death among adults aged ≥60 years hospitalized for RSV than influenza [14], while another showed similar mortality risks among critically-ill patients [15]. This study builds upon the literature by assessing both long- and short-term mortality risks, adjusting for an extensive list of potential confounders, and utilizing comparator groups representing the general population aged ≥50 years and a population experiencing another severe, vaccine-preventable respiratory disease (influenza).

Differences in readmission, all-cause mortality, and MI risks were small in the RSV-ARI and influenza-ARI cohorts, consistent with the literature [13, 16]. A cohort study of US adults aged ≥18 years hospitalized with RSV found that clinical outcomes of RSV were worse or similar to influenza, and influenza severity reduced with vaccination [17]. However, additional reports showed that 46.2% of adults aged 50–64 and 69.7% aged ≥65 years received an influenza vaccination during the 2023–2024 season [18], and only ∼10–25% aged ≥60 years received an RSV vaccine during the 2023–2024 season [19, 20]. This vaccination uptake disparity underscores the potential benefit of an RSV prevention strategy informed by influenza prevention practices.

In evaluating recurrent time-to-event outcomes during follow-up, compared with the influenza-ARI and control cohorts, the RSV-ARI cohort had higher risks of asthma exacerbations, COPD exacerbations, and HHF among patients with those respective comorbidities at baseline. A cohort analysis of hospitalized RSV patients (mean age 75), many with underlying conditions, identified asthma or COPD exacerbations in 27.3% of patients [21]. Another study among adults aged ≥60 years found higher asthma and COPD exacerbation risks for RSV-associated than influenza-associated hospitalization [22]. A cross-sectional study of adults aged ≥50 years hospitalized with RSV identified a high proportion of hospitalizations involving acute cardiac events [23]. By utilizing a control cohort without ARI for adjusted comparisons for the risks of asthma and COPD exacerbations and HHF in people with RSV-ARI, this study offers an improved understanding of the association of these outcomes with severe RSV disease, compared to the background rate expected in people with these comorbidities.

The high clinical burden of RSV-ARI and long-term impacts experienced among hospitalized patients compared with controls is reflected in previous reports of functional status following RSV in older adults. One study found that following RSV hospitalization, adults aged ≥60 years experienced acute functional decline that may become prolonged [6]. RSV-ARI in adults aged ≥65 years has also been associated with long-term reductions in multiple QoL measures (eg, fatigue, social functioning difficulties) [24]. This potential long-term functional status decline mirrors the long-term increased risk of disease exacerbations and other adverse clinical outcomes (eg, MI) in the hospitalized RSV-ARI versus control cohorts during follow-up in our study.

This study highlights similarities between the impact of RSV and influenza, underscoring the importance of improving RSV prevention efforts. These findings could help to inform healthcare provider conversations around RSV, leveraging existing patient awareness of influenza. Results from this study also contribute to the understanding of risks and benefits around RSV vaccination, demonstrating a potentially greater risk of adverse clinical outcomes associated with RSV than previously described. Further investigation into short- and long-term clinical outcomes due to severe RSV disease is needed to inform RSV prevention efforts.

### Limitations

This study may be subject to biases typical of real-world retrospective database analyses. Exact date of death was not available in the Optum database, only month, and therefore was imputed to the 15th of the month, limiting time-to-event mortality analysis precision.

Under-diagnosis of RSV is common [25], possibly leading to selection bias as the study design likely did not capture all eligible patients with RSV; characteristics of patients with undiagnosed RSV may differ from those identified. However, only hospitalized ARI episodes were included, and RSV underdiagnosis may be more prevalent in non-hospitalized settings. ARI episodes with both RSV and influenza diagnoses were classified as RSV-ARI episodes, under the expectation that coinfection was relatively rare (6% in this study) [4, 26], and many episodes following this coding pattern may have represented true RSV-ARI episodes without coinfection but with influenza miscoding. Similarly, identification of comorbidities relied on ICD-10-CM diagnosis codes on medical claims. These claims are submitted for payment and not research, and they may not always accurately reflect patients’ true diagnoses. This could introduce inaccuracies in the measurement of confounding by clinical characteristics and impact stratifications based on comorbidities.

Control cohort patients could not have had a medically-attended RSV- or influenza-ARI episode at age ≥50 years within the analysis period. These criteria may have introduced bias through the selection of healthier-than-average patients with fewer comorbidities, though impacts may have been partially mitigated by limited average duration of enrollment in commercial or Medicare Part D plans. Differences in condition severity and other characteristics unmeasurable using claims data were unable to be accounted for in the adjusted analyses, potentially introducing bias through residual confounding. Therefore, the study did not capture patients’ entire medical histories, likely resulting in inclusion of some control patients with RSV- or influenza-ARI episodes outside data availability. Adjusted analyses also controlled for potential imbalances in baseline characteristics, accounting for measured differences.

Despite overlap of the COVID-19 pandemic (beginning March 2020), the 2015–2023 study period captured periods before, during, and postpandemic, and analyses adjusted for respiratory virus seasonality and COVID-19 temporality by using splines in each regression model. Control patients were matched to the RSV-ARI cohort on index to account for temporal variability. Changes in RSV testing patterns, possibly influenced by the pandemic, including increased outpatient RSV detections post-2020 [27], may have improved generalizability for patients with RSV-ARI identified in later years.

Since RSV vaccines are now available for use in US adults [28], outcomes associated with RSV illness may improve as RSV vaccination uptake continues to increase.

## CONCLUSIONS

Substantial long-term impacts of RSV-ARI hospitalizations among US adults aged ≥50 years were observed in this study. Compared with control and hospitalized influenza-ARI patients, hospitalized RSV-ARI patients showed a greater risk of adverse clinical outcomes including asthma and COPD exacerbations and HHF; risks of all-cause mortality and MI were higher when compared with controls, but generally risks were similar when compared to hospitalized influenza-ARI patients. Severe RSV had a significant impact on clinical outcomes similar to that of influenza, highlighting the potential benefits of RSV prevention beyond avoiding acute disease impacts. These results underscore the need for increased awareness of the clinical burden of RSV among adults aged ≥50 years and the role of RSV prevention in reducing the risk of adverse clinical outcomes.

## Supplementary Material

ciaf710_Supplementary_Data

## Supplementary Data

Supplementary materials are available at *Clinical Infectious Diseases* online. Consisting of data provided by the authors to benefit the reader, the posted materials are not copyedited and are the sole responsibility of the authors, so questions or comments should be addressed to the corresponding author.
