# Supplementary material for: Long-term Clinical Outcomes After Hospitalization for Acute Respiratory Illness Due to Respiratory Syncytial Virus
Source: Clin Infect Dis. 2025 Dec 23;82(6):e1333–44. doi: 10.1093/cid/ciaf710 (PMC13341240; doi:10.1093/cid/ciaf710)
Supplement: ciaf710_Supplementary_Data [file ciaf710_supplementary_data.zip › Supplementary Materials.docx]

SUPPLEMENTARY MATERIALS

### Supplementary Table 1. Study outcomes and definitions

| **Outcomes during the ARI episode** | **Definition** |
| --- | --- |
| Invasive mechanical ventilation use or ICU admission | Binary, invasive mechanical ventilation use or ICU admission identified by the relevant CPT codes and ICD-10-PCS codes for invasive mechanical ventilation use, and the relevant revenue codes for ICU admission |
| Discharge destination from the last inpatient admission during the ARI episode | Binary, discharge to long-term care facilities, skilled nursing facilities, and/or home health services based on discharge codes from inpatient admissions |
| **Outcomes during the entire follow-up period** | **Definition^a^** |
| *Time-to-first event outcomes^b^* | |
| Hospital readmission | Defined among patients with ≥1 inpatient admissions during the ARI episode who were not expired at the time of discharge from the first hospitalization during the ARI episode; the start date for the readmission outcome was the discharge date from the first inpatient admission during the ARI episode |
| All-cause mortality | Defined by death status and month of death; date of death was imputed as the 15^th^ of the month of death |
| MI | ICD-10: I21, I22 |
| *Recurrent time-to-event outcomes^c^* | |
| Exacerbation of asthma [1]^d^ | During an inpatient admission   - Having an asthma exacerbation diagnosis code and no diagnosis code for COPD, acute MI, or HF   During an ED visit   - Having an asthma exacerbation diagnosis code and - Having ≥1 claim for a systemic corticosteroid (with 3–30 days of supply or a single injection of corticosteroids) within ±5 days of the ED visit and - Having no diagnosis codes for COPD, acute MI, HF, or autoimmune diseases during the ED visit   During an OP visit   - Having an asthma exacerbation diagnosis code and - Having ≥1 claim for a systemic corticosteroid (with 3–30 days of supply or a single injection of corticosteroids) within ±5 days of the OP visit and - Having no diagnosis codes for COPD or autoimmune diseases during the OP visit |
| Exacerbation of COPD [2]^d^ | Moderate exacerbation   - An outpatient or ED visit associated with a diagnosis of COPD as a primary or any secondary diagnosis and at least one dispensing for a systemic corticosteroid or antibiotic within seven days of such visit   Severe exacerbation   - An inpatient stay with a claim associated with a diagnosis of COPD as a primary diagnosis |
| Hospitalization due to HF [3] | Defined based on the primary diagnosis code (ICD-10: I50, I11.0, I13.0, I13.2) |

^a^Recurrent time-to-events were defined based on the algorithms listed in the Definition column, consistent with previous studies as cited in Table S1.

^b^Time-to-first events were identified using diagnosis codes on any position in a claim and any type of claim.

^c^Multiple recurrences were allowed and measured for these outcomes.

^d^Assessed overall in all analyses and not stratified by setting or severity.

Abbreviations: ARI, acute respiratory illness; COPD, chronic obstructive pulmonary disease; CPT, current procedural terminology; ED, emergency department; HF, heart failure; ICD-10, International Classification of Diseases, Tenth Revision; ICU, intensive care unit; MI, myocardial infarction; OP, outpatient; PCS, procedural coding system.

### Supplementary Table 2. Subgroup analyses conducted for time-to-first-event outcomes

| **Subgroup** | **Outcomes** | | |
| --- | --- | --- | --- |
|  | **Hospital readmission** | **MI** | **All-cause mortality** |
| **Overall** | X | X | X |
| **Age group (50–59, 60–64, 65–74, ≥75 years)** | X | X | X |
| **Comorbidity** |  |  |  |
| Any chronic condition |  |  | X |
| Chronic respiratory diseases |  | X |  |
| Chronic cardiovascular diseases |  | X |  |
| COPD | X |  | X |
| Asthma | X |  | X |
| HF | X | X | X |
| CAD | X | X | X |
| Diabetes | X | X | X |

Subgroup analyses of interest are indicated with an “X”.

Abbreviations: CAD, coronary artery disease; COPD, chronic obstructive pulmonary disease; HF, heart failure; MI, myocardial infarction.

### Supplementary Table 3. Subgroup analyses conducted for recurrent event outcomes

| **Subgroups** | **Outcomes** | | |
| --- | --- | --- | --- |
|  | **Asthma exacerbation** | **COPD exacerbation** | **HHF** |
| **Overall** | X | X | X |
| **Age group (50–59, 60–64, 65–74, ≥75 years)** | X | X | X |
| **Comorbidity** |  |  |  |
| Chronic cardiovascular diseases |  |  | X |
| COPD |  | X |  |
| Asthma | X |  |  |
| HF |  |  | X |

Subgroup analyses of interest are indicated with an “X”. COPD exacerbation, asthma exacerbation, and hospitalization due to HF were assessed in the overall population as well as those with evidence of COPD, asthma, and HF during baseline. It was likely that some patients who had asthma, COPD, or HF prior to index did not have claims for COPD, asthma, and HF during baseline but experienced exacerbations of existing disease during the follow-up period. Such patients were included in the analysis for the overall population but not the analysis among patients who had COPD, asthma, and HF during baseline.

Abbreviations: COPD, chronic obstructive pulmonary disease; HHF, hospitalization due to heart failure; HF, heart failure.

### Supplementary Figure 1. Sample selection flowchart


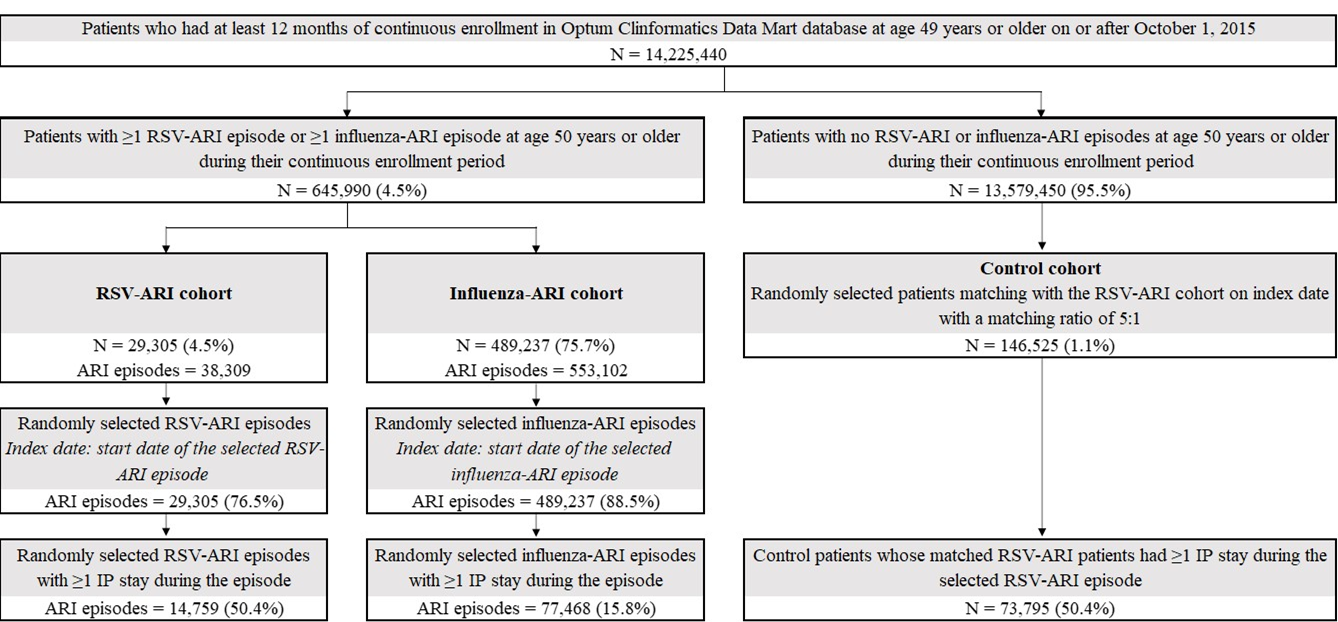


Abbreviations: ARI, acute respiratory illness; IP, inpatient; RSV, respiratory syncytial virus.

### Supplementary Table 4. Outcomes during ARI episodes

| **Outcome** | **RSV-ARI Cohort**  **(N=14,759)** | **Influenza-ARI Cohort**  **(N=77,468)** |
| --- | --- | --- |
| Duration of ARI episode in days (mean ± SD) | 22.76 ± 27.56 | 21.90 ± 31.50 |
| Invasive mechanical ventilation use or ICU admission (n [%]) | 1,155 (7.83%) | 6,785 (8.76%) |
| Discharge destination (n [%])  Long-term care facility^a^  Skilled nursing facility^a^  Home health services^a^ | 13 (0.09%)  425 (2.98%)  3,030 (21.26%) | 75 (0.10%)  2,071 (2.77%)  14,253 (19.04%) |

^a^Discharge destination outcomes were assessed among the 14,249 patients in the RSV-ARI cohort and 74,877 patients in the influenza-ARI cohort who were discharged alive from the last IP admission during their selected ARI episode.

Abbreviations: ARI, acute respiratory illness; ICU, intensive care unit; RSV, respiratory syncytial disease; SD, standard deviation.

### Supplementary Table 5. Risk of hospital readmission at 30 days and 3 months by age group

|  | | | **Risk of hospital readmission^a^ at key time points** | |
| --- | --- | --- | --- | --- |
| **Cohort** | **Number of patients** |  | **30 days** | **3 months** |
| **Age 50–59 years** | | | | |
| RSV-ARI with hospitalization | 927 | Number at risk | 729 | 580 |
|  |  | Event probability^b^ (95% CI) | 0.162 (0.139, 0.187) | 0.282 (0.253, 0.312) |
|  |  |  |  |  |
| Influenza-ARI with hospitalization | 6,047 | Number at risk | 4,980 | 4,184 |
|  |  | Event probability^b^ (95% CI) | 0.119 (0.111, 0.127) | 0.214 (0.204, 0.225) |
|  |  |  |  |  |
| **Age 60–64 years** | | | | |
| RSV-ARI with hospitalization | 942 | Number at risk | 710 | 574 |
|  |  | Event probability^b^ (95% CI) | 0.181 (0.157, 0.207) | 0.293 (0.264, 0.323) |
|  |  |  |  |  |
| Influenza-ARI with hospitalization | 5,561 | Number at risk | 4,381 | 3,682 |
|  |  | Event probability^b^ (95% CI) | 0.147 (0.138, 0.157) | 0.238 (0.226, 0.249) |
|  |  |  |  |  |
| **Age 65–74 years** | | | | |
| RSV-ARI with hospitalization | 3,890 | Number at risk | 3,005 | 2,442 |
|  |  | Event probability^b^ (95% CI) | 0.160 (0.149, 0.172) | 0.270 (0.256, 0.284) |
|  |  |  |  |  |
| Influenza-ARI with hospitalization | 21,335 | Number at risk | 16,774 | 14,158 |
|  |  | Event probability^b^ (95% CI) | 0.145 (0.140, 0.150) | 0.233 (0.227, 0.238) |
|  |  |  |  |  |

| **Age ≥75 years** | | | | |
| --- | --- | --- | --- | --- |
| RSV-ARI with hospitalization | 8,498 | Number at risk | 6,253 | 5,098 |
|  |  | Event probability^b^ (95% CI) | 0.159 (0.151, 0.167) | 0.250 (0.241, 0.260) |
|  |  |  |  |  |
| Influenza-ARI with hospitalization | 41,987 | Number at risk | 31,585 | 26,178 |
|  |  | Event probability^b^ (95% CI) | 0.146 (0.143, 0.150) | 0.236 (0.232, 0.240) |
|  |  |  |  |  |

^a^Readmission was evaluated among patients in the RSV-ARI and influenza-ARI cohorts who had inpatient as the highest level of care in the selected ARI episode. Patients who died during the first ARI episode hospitalization were excluded. The start date for the readmission outcome was the discharge date of the first hospitalization during the ARI episode.
^b^The probability of hospital readmission was estimated based on the cumulative incidence function, accounting for death as a competing risk.

Abbreviations: ARI, acute respiratory illness; CI, confidence interval; RSV, respiratory syncytial virus.

### Supplementary Table 6. Risk of hospital readmission at 30 days and 3 months by baseline comorbidity

|  | | | **Risk of hospital readmission^a^ at key time points** | |  |
| --- | --- | --- | --- | --- | --- |
| **Cohort** | **Number of patients** |  | **30 days** | **3 months** |  |
| **Diabetes** | | | | |  |
| RSV-ARI with hospitalization | 6,154 | Number at risk | 4,472 | 3,518 |  |
|  |  | Event probability^b^ (95% CI) | 0.186 (0.176, 0.195) | 0.304 (0.292, 0.315) |  |
|  |  |  |  |  |  |
| Influenza-ARI with hospitalization | 33,101 | Number at risk | 24,840 | 20,154 |  |
|  |  | Event probability^b^ (95% CI) | 0.165 (0.161, 0.169) | 0.270 (0.265, 0.275) |  |
|  |  |  |  |  |  |
| **COPD** | | | | |  |
| RSV-ARI with hospitalization | 6,471 | Number at risk | 4,637 | 3,668 |  |
|  |  | Event probability^b^ (95% CI) | 0.190 (0.181, 0.200) | 0.303 (0.292, 0.315) |  |
|  |  |  |  |  |  |
| Influenza-ARI with hospitalization | 28,568 | Number at risk | 20,907 | 16,654 |  |
|  |  | Event probability^b^ (95% CI) | 0.179 (0.174, 0.183) | 0.289 (0.284, 0.295) |  |
|  |  |  |  |  |  |
| **Asthma** | | | | |  |
| RSV-ARI with hospitalization | 2,691 | Number at risk | 2,030 | 1,636 |  |
|  |  | Event probability^b^ (95% CI) | 0.171 (0.157, 0.186) | 0.287 (0.270, 0.305) |  |
|  |  |  |  |  |  |
| Influenza-ARI with hospitalization | 11,274 | Number at risk | 8,628 | 7,063 |  |
|  |  | Event probability^b^ (95% CI) | 0.166 (0.159, 0.173) | 0.272 (0.264, 0.280) |  |
|  |  |  |  |  |  |

| **Heart failure** | | | | |  |
| --- | --- | --- | --- | --- | --- |
| RSV-ARI with hospitalization | 5,485 | Number at risk | 3,706 | 2,773 |  |
|  |  | Event probability^b^ (95% CI) | 0.220 (0.209, 0.231) | 0.353 (0.340, 0.366) |  |
|  |  |  |  |  |  |
| Influenza-ARI with hospitalization | 23,053 | Number at risk | 15,918 | 12,060 |  |
|  |  | Event probability^b^ (95% CI) | 0.205 (0.200, 0.210) | 0.333 (0.327, 0.339) |  |
|  |  |  |  |  |  |
| **CAD** | | | | |  |
| RSV-ARI with hospitalization | 5,766 | Number at risk | 4,071 | 3,140 |  |
|  |  | Event probability^b^ (95% CI) | 0.196 (0.186, 0.207) | 0.317 (0.305, 0.330) |  |
|  |  |  |  |  |  |
| Influenza-ARI with hospitalization | 27,291 | Number at risk | 19,936 | 15,749 |  |
|  |  | Event probability^b^ (95% CI) | 0.177 (0.172, 0.181) | 0.292 (0.286, 0.297) |  |
|  |  |  |  |  |  |

^a^Readmission was evaluated among patients in the RSV-ARI and influenza-ARI cohorts who had inpatient as the highest level of care in the selected ARI episode. Patients who died during the first ARI episode hospitalization were excluded. The start date for the readmission outcome was the discharge date of the first hospitalization during the ARI episode.
^b^The probability of hospital readmission was estimated based on the cumulative incidence function, accounting for death as a competing risk.

Abbreviations: ARI, acute respiratory illness; CAD, coronary artery disease; CI, confidence interval; COPD, chronic obstructive pulmonary disease; RSV, respiratory syncytial virus.

### Supplementary Table 7. Risk of MI at key time points by age group

|  | | | **Risk of MI at key time points** | | | | |  |
| --- | --- | --- | --- | --- | --- | --- | --- | --- |
| **Cohort** | **Number of patients** |  | **30 days** | **3 months** | **12 months** | **3 years** | **5 years** |  |
| **Age 50–59 years** | | | | | | | |  |
| RSV-ARI with hospitalization | 945 | Number at risk | 836 | 761 | 445 | 168 | 46 |  |
|  |  | Event probability^1^  (95% CI) | 0.070  (0.055, 0.087) | 0.077  (0.061, 0.095) | 0.116  (0.096, 0.138) | 0.168  (0.139, 0.200) | 0.197  (0.160, 0.238) |  |
| Influenza-ARI with hospitalization | 6,149 | Number at risk | 5,433 | 4,989 | 3,261 | 1,602 | 472 |  |
|  |  | Event probability^1^  (95% CI) | 0.072  (0.066, 0.079) | 0.081  (0.075, 0.089) | 0.105  (0.098, 0.114) | 0.144  (0.134, 0.154) | 0.181  (0.167, 0.195) |  |
| Control | 15,372 | Number at risk | 14,937 | 14,187 | 9,176 | 4,539 | 1,291 |  |
|  |  | Event probability^1^  (95% CI) | 0.001  (0.000, 0.001) | 0.001  (0.001, 0.002) | 0.006  (0.005, 0.007) | 0.018  (0.016, 0.022) | 0.031  (0.026, 0.036) |  |
| **Age 60–64 years** | | | | | | | |  |
| RSV-ARI with hospitalization | 966 | Number at risk | 832 | 741 | 399 | 159 | 40 |  |
|  |  | Event probability^1^  (95% CI) | 0.086  (0.069, 0.105) | 0.101  (0.083, 0.121) | 0.140  (0.118, 0.164) | 0.185  (0.156, 0.217) | 0.218  (0.179, 0.260) |  |
| Influenza-ARI with hospitalization | 5,703 | Number at risk | 4,903 | 4,420 | 2,787 | 1,259 | 353 |  |
|  |  | Event probability^1^  (95% CI) | 0.085  (0.078, 0.092) | 0.096  (0.088, 0.104) | 0.124  (0.115, 0.133) | 0.174  (0.163, 0.186) | 0.220  (0.203, 0.236) |  |
| Control | 7,828 | Number at risk | 7,613 | 7,196 | 4,568 | 1,925 | 481 |  |
|  |  | Event probability^1^  (95% CI) | 0.002  (0.001, 0.003) | 0.004  (0.003, 0.005) | 0.011  (0.009, 0.014) | 0.026  (0.022, 0.032) | 0.042  (0.034, 0.051) |  |
| **Age 65–74 years** | | | | | | | |  |
| RSV-ARI with hospitalization | 3,986 | Number at risk | 3,444 | 3,053 | 1,733 | 777 | 193 |  |
|  |  | Event probability^1^  (95% CI) | 0.090  (0.081, 0.099) | 0.105  (0.095, 0.114) | 0.141  (0.130, 0.152) | 0.203  (0.188, 0.219) | 0.236  (0.218, 0.254) |  |
| Influenza-ARI with hospitalization | 21,955 | Number at risk | 18,508 | 16,727 | 10,831 | 5,785 | 1,962 |  |
|  |  | Event probability^1^  (95% CI) | 0.104  (0.100, 0.108) | 0.114  (0.110, 0.118) | 0.143  (0.138, 0.147) | 0.191  (0.185, 0.196) | 0.229  (0.222, 0.237) |  |
| Control | 26,094 | Number at risk | 25,568 | 24,507 | 16,064 | 9,367 | 2,867 |  |
|  |  | Event probability^1^  (95% CI) | 0.002 (0.001, 0.002) | 0.005 (0.004, 0.006) | 0.014 (0.013, 0.016) | 0.036 (0.033, 0.039) | 0.057 (0.052, 0.062) |  |
| **Age ≥75 years** | | | | | | | |  |
| RSV-ARI with hospitalization | 8,862 | Number at risk | 7,172 | 6,151 | 3,527 | 1,487 | 339 |  |
|  |  | Event probability^1^  (95% CI) | 0.110  (0.103, 0.116) | 0.120  (0.114, 0.127) | 0.150  (0.142, 0.158) | 0.194  (0.185, 0.203) | 0.222  (0.211, 0.233) |  |
| Influenza-ARI with hospitalization | 43,661 | Number at risk | 35,551 | 31,162 | 20,398 | 9,775 | 3,244 |  |
|  |  | Event probability^1^  (95% CI) | 0.109  (0.106, 0.112) | 0.118  (0.115, 0.121) | 0.146  (0.142, 0.149) | 0.187  (0.183, 0.191) | 0.214  (0.210, 0.219) |  |
| Control | 24,501 | Number at risk | 24,011 | 22,888 | 14,816 | 8,118 | 2,288 |  |
|  |  | Event probability^1^  (95% CI) | 0.004  (0.003, 0.004) | 0.008  (0.007, 0.009) | 0.024  (0.022, 0.027) | 0.060  (0.057, 0.064) | 0.098  (0.092, 0.104) |  |

^a^The probability of MI was estimated based on the cumulative incidence function, accounting for death as a competing risk.

Abbreviations: ARI, acute respiratory illness; CI, confidence interval; MI, myocardial infarction; RSV, respiratory syncytial virus.

### Supplementary Table 8. Adjusted association of MI with RSV infection using Cox proportional hazards models with time-varying coefficients among age subgroups^a,b^

| **MI** | **Age 50–59 years** | | **Age 60–64 years** | | **Age 65–74 years** | | **Age ≥75 years** | |
| --- | --- | --- | --- | --- | --- | --- | --- | --- |
|  | **Hazard ratio (95% CI)** | ***P*-value** | **Hazard ratio (95% CI)** | ***P*-value** | **Hazard ratio (95% CI)** | ***P*-value** | **Hazard ratio (95% CI)** | ***P*-value** |
| **RSV cohort vs. Influenza cohort** | | | | |  |  |  |  |
| Period of 0–30 days | 0.863 | 0.277 | 0.863 | 0.226 | 0.765*** | <0.001 | 0.923* | 0.027 |
|  | (0.661–1.126) |  | (0.679–1.096) |  | (0.683–0.858) |  | (0.860–0.991) |  |
| Period of 31–90 days | 0.659 | 0.335 | 1.180 | 0.578 | 1.294 | 0.090 | 1.034 | 0.779 |
|  | (0.283–1.537) |  | (0.658–2.117) |  | (0.960–1.745) |  | (0.821–1.301) |  |
| Period of 91–365 days | 1.547* | 0.035 | 1.217 | 0.352 | 1.200 | 0.077 | 1.052 | 0.504 |
|  | (1.031–2.321) |  | (0.805–1.838) |  | (0.981–1.470) |  | (0.907–1.219) |  |
| Period >365 days | 1.175 | 0.448 | 0.954 | 0.827 | 1.258** | 0.008 | 1.075 | 0.248 |
|  | (0.775–1.783) |  | (0.628–1.450) |  | (1.063–1.488) |  | (0.951–1.214) |  |
| **RSV cohort vs. Control cohort** | | | | |  |  |  |  |
| Period of 0–30 days | 59.778*** | <0.001 | 29.490*** | <0.001 | 34.481*** | <0.001 | 24.871*** | <0.001 |
|  | (30.473–117.266) |  | (16.298–53.360) |  | (25.302–46.989) |  | (19.969–30.976) |  |
| Period of 31–90 days | 6.091*** | <0.001 | 4.973*** | <0.001 | 3.842*** | <0.001 | 2.488*** | <0.001 |
|  | (2.201–16.857) |  | (2.385–10.367) |  | (2.702–5.464) |  | (1.867–3.316) |  |
| Period of 91–365 days | 6.502*** | <0.001 | 3.995*** | <0.001 | 3.383*** | <0.001 | 2.087*** | <0.001 |
|  | (4.097–10.319) |  | (2.465–6.474) |  | (2.685–4.262) |  | (1.755–2.483) |  |
| Period >365 days | 3.339*** | <0.001 | 2.940*** | <0.001 | 3.081*** | <0.001 | 1.672*** | <0.001 |
|  | (2.155–5.172) |  | (1.851–4.671) |  | (2.566–3.701) |  | (1.461–1.912) |  |

**P*<0.05, ***P*<0.01, ****P*<0.001

^a^Cox proportional hazards models with time-varying coefficients were used to estimate the hazard ratios.

^b^The analysis was conducted among patients in the combined sample of RSV-ARI cohort, influenza-ARI cohort, and control cohort. Patients in the RSV-ARI and influenza-ARI cohorts were restricted to those with IP as the highest level of care in their selected ARI episode. Patients in the control cohort were restricted to those with their matched RSV patients having IP as the highest level of care in the selected ARI episode.

Abbreviations: ARI, acute respiratory illness; CI, confidence interval; IP, inpatient; MI, myocardial infarction; RSV, respiratory syncytial virus; vs, versus.

### Supplementary Table 9. Risk of MI at key time points by baseline comorbidity

|  | | | **Risk of MI at key time points** | | | | |  |
| --- | --- | --- | --- | --- | --- | --- | --- | --- |
| **Cohort** | **Number of patients** |  | **30 days** | **3 months** | **12 months** | **3 years** | **5 years** |  |
| **Diabetes** | | | | | | | |  |
| RSV-ARI with hospitalization | 6,379 | Number at risk | 5,242 | 4,514 | 2,505 | 1,015 | 220 |  |
|  |  | Event probability^a^  (95% CI) | 0.115  (0.107, 0.123) | 0.130  (0.122, 0.139) | 0.173  (0.164, 0.183) | 0.234  (0.222, 0.246) | 0.267  (0.253, 0.282) |  |
| Influenza-ARI with hospitalization | 34,253 | Number at risk | 28,099 | 24,827 | 15,731 | 7,377 | 2,271 |  |
|  |  | Event probability^a^  (95% CI) | 0.116  (0.112, 0.119) | 0.128  (0.124, 0.132) | 0.163  (0.159, 0.167) | 0.217  (0.213, 0.222) | 0.255  (0.249, 0.261) |  |
| Control | 17,595 | Number at risk | 17,154 | 16,296 | 10,420 | 5,488 | 1,529 |  |
|  |  | Event probability^a^  (95% CI) | 0.005  (0.004, 0.006) | 0.010  (0.009, 0.012) | 0.028  (0.026, 0.031) | 0.070  (0.065, 0.075) | 0.109  (0.101, 0.117) |  |
| **Heart failure** | | | | | | | |  |
| RSV-ARI with hospitalization | 5,746 | Number at risk | 4,577 | 3,824 | 1,989 | 696 | 138 |  |
|  |  | Event probability^a^  (95% CI) | 0.123  (0.115, 0.132) | 0.141  (0.132, 0.150) | 0.190  (0.179, 0.200) | 0.251  (0.238, 0.264) | 0.272  (0.258, 0.287) |  |
| Influenza-ARI with hospitalization | 24,067 | Number at risk | 19,034 | 16,198 | 9,361 | 3,727 | 1,017 |  |
|  |  | Event probability^a^  (95% CI) | 0.132  (0.128, 0.137) | 0.149  (0.144, 0.153) | 0.194  (0.189, 0.199) | 0.251  (0.245, 0.257) | 0.280  (0.273, 0.286) |  |
| Control | 5,120 | Number at risk | 4,918 | 4,545 | 2,661 | 1,182 | 269 |  |
|  |  | Event probability^a^  (95% CI) | 0.015  (0.012, 0.019) | 0.026  (0.022, 0.030) | 0.064  (0.057, 0.071) | 0.123  (0.111, 0.135) | 0.179  (0.162, 0.197) |  |
| **CAD** | | | | | | | |  |
| RSV-ARI with hospitalization | 6,000 | Number at risk | 4,766 | 4,030 | 2,173 | 835 | 169 |  |
|  |  | Event probability^a^  (95% CI) | 0.134  (0.125, 0.142) | 0.154  (0.145, 0.163) | 0.202  (0.191, 0.212) | 0.267  (0.254, 0.280) | 0.295  (0.281, 0.310) |  |
| Influenza-ARI with hospitalization | 28,374 | Number at risk | 22,550 | 19,554 | 11,957 | 5,189 | 1,499 |  |
|  |  | Event probability^a^  (95% CI) | 0.138  (0.134, 0.142) | 0.154  (0.150, 0.158) | 0.200  (0.195, 0.204) | 0.261  (0.255, 0.267) | 0.299  (0.293, 0.306) |  |
| Control | 10,589 | Number at risk | 10,241 | 9,633 | 5,982 | 2,997 | 762 |  |
|  |  | Event probability^a^  (95% CI) | 0.011  (0.009, 0.013) | 0.023  (0.021, 0.026) | 0.059  (0.054, 0.064) | 0.119  (0.111, 0.127) | 0.178  (0.166, 0.190) |  |
| **Chronic cardiac diseases** | | | | | | | |  |
| RSV-ARI with hospitalization | 9,602 | Number at risk | 7,782 | 6,634 | 3,643 | 1,429 | 296 |  |
|  |  | Event probability^a^  (95% CI) | 0.116  (0.109, 0.122) | 0.130  (0.124, 0.137) | 0.171  (0.163, 0.179) | 0.227  (0.217, 0.236) | 0.254  (0.243, 0.265) |  |
| Influenza-ARI with hospitalization | 44,218 | Number at risk | 35,807 | 31,191 | 19,264 | 8,615 | 2,600 |  |
|  |  | Event probability^a^  (95% CI) | 0.119  (0.116, 0.122) | 0.133  (0.130, 0.136) | 0.170  (0.166, 0.174) | 0.223  (0.219, 0.228) | 0.258  (0.253, 0.263) |  |
| Control | 17,289 | Number at risk | 16,785 | 15,851 | 9,813 | 4,928 | 1,282 |  |
|  |  | Event probability^a^  (95% CI) | 0.008  (0.006, 0.009) | 0.016  (0.014, 0.018) | 0.044  (0.041, 0.047) | 0.097  (0.091, 0.103) | 0.146  (0.137, 0.155) |  |
| **Chronic respiratory diseases** | | | | | | | |  |
| RSV-ARI with hospitalization | 8,396 | Number at risk | 7,019 | 6,072 | 3,430 | 1,423 | 337 |  |
|  |  | Event probability^a^  (95% CI) | 0.094  (0.088, 0.101) | 0.108  (0.102, 0.115) | 0.145  (0.137, 0.153) | 0.199  (0.189, 0.209) | 0.229  (0.217, 0.241) |  |
| Influenza-ARI with hospitalization | 37,344 | Number at risk | 31,111 | 27,343 | 17,263 | 8,115 | 2,496 |  |
|  |  | Event probability^a^  (95% CI) | 0.101  (0.098, 0.104) | 0.113  (0.110, 0.116) | 0.146  (0.142, 0.150) | 0.196  (0.191, 0.200) | 0.230  (0.225, 0.235) |  |
| Control | 11,685 | Number at risk | 11,355 | 10,715 | 6,817 | 3,494 | 949 |  |
|  |  | Event probability^a^  (95% CI) | 0.005  (0.003, 0.006) | 0.011  (0.009, 0.013) | 0.029  (0.026, 0.032) | 0.067  (0.061, 0.073) | 0.109  (0.099, 0.119) |  |

^a^The event probability was estimated based on the cumulative incidence function, accounting for death as a competing risk.

Abbreviations: ARI, acute respiratory illness; CAD, coronary artery disease; CI, confidence interval; MI, myocardial infarction; RSV, respiratory syncytial virus.

**Supplement References**

1. Chung Y, Katial R, Mu F, Cook EE, Young J, Yang D, Betts KA, Carstens DD. Real-world effectiveness of benralizumab: Results from the ZEPHYR 1 Study. Ann Allergy Asthma Immunol **2022**; 128(6): 669-676.e666.

2. Mannino D, Weng S, Germain G, Boudreau J, Tardif-Samson A, Forero-Schwanhaeuser S, Laliberté F, Gravelle P, Compton CH, Noorduyn SG, Paczkowski R. Comparative Effectiveness of Fluticasone Furoate/Umeclidinium/Vilanterol and Budesonide/Glycopyrrolate/Formoterol Fumarate among US Patients with Chronic Obstructive Pulmonary Disease. Adv Ther **2025**; 42(2): 1131-1146.

3. Zhang M, Solomon DH, Desai RJ, Kang EH, Liu J, Neogi T, Kim SC. Assessment of Cardiovascular Risk in Older Patients With Gout Initiating Febuxostat Versus Allopurinol: Population-Based Cohort Study. Circulation **2018**; 138(11): 1116-1126.
